# Deep-water bivalve mollusks collected during the TALUD XV cruise off the west coast of the southern Baja California Peninsula, Mexico 

**DOI:** 10.3897/BDJ.4.e8661

**Published:** 2016-05-20

**Authors:** Michel E. Hendrickx, Paul Valentich-Scott, Nancy Yolimar Suárez-Mozo

**Affiliations:** ‡ Instituto de Ciencias del Mar y Limnología, Universidad Nacional Autónoma de México, Mazatlán, Mexico; § Santa Barbara Museum of Natural History, Santa Barbara, United States of America; | Posgrado en Ciencias del Mar y Limnologia, Universidad Nacional Autónoma de México, Mazatlán, Mexico; ¶ Laboratorio de Invertebrados Bentónicos, Mazatlán, Mexico

**Keywords:** Mollusks, Bivalvia, deep-water, continental slope, western Baja California, Mexico

## Abstract

**Background:**

During the TALUD XV research cruise off the southern part of the Baja California Peninsula, Mexico, samples of macro-invertebrates obtained in the deep-sea (296–2136 m) revealed a rich fauna of bivalves (17 species belonging to 10 families). The number of species per station varied from one to five. The richest families were Nuculidae, Nuculanidae, Neilonellidae, Limidae, and Cuspidariidae. Solemyidae, Lucinidae, Poromyidae, Verticordiidae, and Pectinidae were each represented by a single species. Some species groups need a thorough revision and were tentatively identified (Nuculana cf. hamata, Limatula cf. saturna).

**New information:**

Significant new distribution information is provided for two species, both recorded for the first time from off western Mexico: *Ennucula panamina* with an extension of its known distribution over 20° of latitude north and *Jupiteria callimene* with an extension of 16° 42' of latitude to the north. One species (*Ennucula taeniolata*) is reported in shallower depth and one in deeper water (*Acesta sphoni*). New records are provided for an additional nine species. Environmental and habitat conditions are given for the first time for many of the bivalve species.

## Introduction

An intensive survey of deep-water invertebrates in Pacific Mexico has long been overdue (see Zamorano and Hendrickx 2012). Deep-water mollusks collected in the 19th and 20th centuries during the "Albatross" and the "Velero IV" cruises represent the largest collection available to date in this area (see Dall 1895, Emerson and Puffer 1957, Parker 1963).

In her monumental monograph, Keen (1971) reported 792 + 11 = 803 species of "Pelecypoda" from tropical West America, corresponding to what the author called the "Panamic fauna." Keen (1971) defined the survey area as extending from Magdalena Bay, along the SW coast of the Baja California Peninsula, to Punta Aguja, in northern Peru. She included in this area the Galapagos Archipelago and other nearer, offshore islands. Since its publication, this monograph has remained the basic reference book for all species of bivalves and other marine mollusks occurring in the tropical West America. Many taxonomic changes, new species, and new records of bivalves have since been added to the Panamic fauna as described by Keen (1971), most of which were compiled in the contributions of Skoglund (1991), Skoglund (2001).

The recent monograph on marine bivalve mollusks of tropical West America by Coan and Valentich-Scott (2012) provided a new tool for the study of these organisms in the region. It details nearly 900 species, thus representing an increase in bivalve species of about 12% compared to Keen (1971) monograph, and includes the description of several new genera and species.

In 1989, an intensive survey of the deep-water fauna of the Mexican Pacific (the TALUD project) was initiated by the Laboratorio de Invertebrados Bentónicos, Instituto de Ciencias del Mar y Limnología (ICML), Universidad Nacional Autónoma de México (UNAM), in Mazatlán, Mexico. Starting in 2000, sampling was aimed at collecting specimens living below the Oxygen Minimum Zone (OMZ) core (see Hendrickx 2012). In this contribution we report on material collected off the west coast of Baja California during the TALUD XV cruise.

## Materials and Methods

The material reported herein includes both living and empty shells and was collected during the TALUD XV research cruise (29 July to 6 August 2012) aboard the R/V "El Puma" of the UNAM, roughly between 23°05’ N and 27°08’ N off the west coast of southern Baja California. A total of 23 stations were sampled between 296 and 2123 m depth. Specimens of deep–water organisms were collected with a 2.35 m wide by 0.95 m high, standard benthic sledge (Hendrickx 2012) equipped with an outer collecting net of ca 5.5 cm (2 1/4") stretch mesh and an inner net of ca 2.0 cm (3/4") stretch mesh. Trawling generally lasted 30 minutes (some trawls were interrupted after 15 minutes) at an average speed of 1.75 knots. A total of 19 infaunal samples were also obtained using a 40 cm x 40 cm box core (USNEL) in depths from 338 to 2290 m. Sampling depths were estimated with a digital SIMRAD echo sounder. Near bottom environmental parameters were obtained with a Seabird–19 CTD (temperature, T; salinity, S) and dissolved oxygen concentrations (DO) were measured by the Winkler method using water collected with 10 liters Niskin bottles. Sediments (sed.) from an USNEL box core were used for granulometric analysis. For each species the following information is provided: 1) list of material examined; 2) type locality; 3) current geographic distribution; 4) environmental conditions near bottom; 5) remarks, if any. The taxonomic sequence follows Coan and Valentich-Scott (2012). New records from off the west coast of the Baja California Peninsula were based partly on the database of the SCRIPPS Institution of Oceanography mollusks collection and on a large data base containing records of species for this area referred to by Zamorano and Hendrickx (2011), and on information provided by Coan and Valentich-Scott (2012). All the specimens examined are deposited in the Regional Collection of Marine Invertebrates at the Mazatlán Marine Station, UNAM, in Mazatlán, Mexico (ICML–EMU), or at the Santa Barbara Museum of Natural History (SBMNH), Santa Barbara, California, USA. Abbreviations used herein are: sb, scale bar; St., sampling station; Sa, sand; Si, silt; Cl, clay.

## Data resources

This study is based on material collected during the TALUD XV cruise kept in the holding of the Regional Collection of Marine Invertebrates, Mazatlán, Mexico, on pertinent literature and on comprative material available at the Santa Barbara Museum of Natural History, California, USA.

## Results

Specimens of bivalves were found in 10 out of the 23 samples obtained during the survey, between 236 and 2136 m depth. In total, 76 live specimens and 8 empty specimens were collected. The collection contained 18 species in 14 genera belonging to 11 families (Figs 1, 2, 3, 4).

**Systematic section**

In this contribution, we present the species collected during the TALUD XV cruise. The following sections were included: material examined, type locality, distribution of the material collected, general geographic distribution, environmental conditions and remarks. The taxonomic organization follows Coan and Valentich-Scott (2012).

**Clase Bivalvia**

**Order Nuculida**

**Superfamily Nuculoidea**

**Family Nuculidae Gray, 1824**

***Ennucula panamina* (Dall, 1908)**

**Fig. 1a**

**Material examined**. St. 23 (27° 08' 11" N, 114° 32' 54" W), August 1, 2012, 5 specimens, 530–625 m, benthic sledge (ICML–EMU–10973, 1 dry specimen; ICML–EMU–10974, 4 specimens in ethanol.

**Questionable material**. St. 20 (26° 30' 42" N, 113° 56' W), August 2, 2015, 14 specimens (and one empty specimen), 540–568 m depth, benthic sledge (ICML–EMU–9974).

**Type locality**. SW of Isla Coiba, Panama (Albatross St. 3360).

**Distribution**. Off Panama (type locality) and off Punta Mancora, Peru. 550–3,058 m (Parker 1963, Coan and Valentich-Scott 2012).

**Environmental conditions**. DO, 0.068 ml/l; T, 6.44°C; S, 34.47; sed., 46.62% Sa, 46.56% Si, 6.82% Cl.

**Remarks**. *Ennucula panamina* had previously been reported from its type locality (Hertlein and Strong 1940) and from off Punta Mancora (Coan and Valentich-Scott 2012). The record of Parker (1963), in a sample taken below 1000 m depth, was omitted by Coan and Valentich-Scott (2012) as the illustrated specimen was actually *Ennucula cardara* (Dall, 1916). This record is the first for *E. panamina* off western Mexico, represents a new locality for this species, and extends its known distribution over 20° of latitude north of previous reports.

Specimens from St. 20 have proved to be perplexing. We have compared them to the syntype of *E. panamina*, the syntype of *E. taeniolata*, and to illustrations provided by Coan and Valentich-Scott (2012). The material from St. 20 (14 specimens) is closer to the former species, which is characterized by a subtrigonal shell () vs. an elongate-subtrigonal shell in *E. taeniolata* (). However, number of teeth in the anterior hinge of these 14 specimens does not fit well with either *E. taeniolata* (10-11) or with *E. panamina* (20-22) (Coan and Valentich-Scott 2012). Indeed, there are 10-14 anterior teeth, with 10 specimens possessing >11 teeth. In the case of the posterior hinge, however, number of teeth observed in these 14 specimens (6-8) is closer to *E. taeniolata* (i.e., 6-7) than to *E. panamina* (i.e., 10) (Coan and Valentich-Scott 2012). For these reasons, this material is considered doubtful pending further analysis.

***Ennucula taeniolata* (Dall, 1908)**

**Fig. 1b**

**Material examined**. St. 23 (27° 08' 11" N, 114° 32' 54" W), August 1, 2012, 1 specimen in ethanol, 530–625 m, SBMNH 235539.

**Type locality**. Off Acapulco, Guerrero, Mexico (Albatross St. 3417).

**Distribution.** Gulf of California north of Isla Tortuga, Baja California Sur to south of Acapulco, Guerrero, Mexico. 540–1,275 m (this contribution; Coan and Valentich-Scott 2012).

**Environmental conditions.** DO, 0.15 ml/l; T, 8.38° C; S, 34.51; sed., 47.08% Sa, 45.16% Si, 7.75% Cl.

**Remarks.** This is the first record of *Ennucula taeniolata* from the outer coast of Baja California, and represents the shallowest record for this species.

***Ennucula tenuis* (Montagu, 1808)**

Fig. 1c

**Material examined.** St. 24 (27° 05' 42" N, 114° 35' 30" W), August 1, 2015, 1 specimen, 772–786 m depth (ICML–EMU–9975).

**Type locality.** Dumbar, England.

**Distribution.** Throughout the Bering Sea and the Gulf of Alaska, and San Diego, California, USA to Isla Cedros, Southern Gulf of California, Mexico, and to Punta Guiones, Guanacaste, Costa Rica; Also reported from the Mediterranean, Florida and northern Japan. 201–450 m (Zamorano et al. 2007, Coan and Valentich-Scott 2012).

**Environmental conditions.** DO, 0.12 ml/l; T, 5.24° C; S, 34.53; sed., 35.53% Sa, 56.52% Si, 7.95% Cl.

**Remarks.** Based on its wide geographic and bathymetric distribution, *E. tenuis* likely represents a large species complex.

**Order Solemyida**

**Superfamily Solemyoidea**

**Family Solemyidae Gray, 1840**

***Acharax johnsoni* (Dall, 1891)**

Fig. 1e

**Material examined.** St. 5D (23° 16' 58" N, 110° 20' 42" W), August 5, 2012, 5 empty specimens, 650–665 m, benthic sledge (ICML–EMU–9976).

**Type locality.** Off Lower California Coast (Albatross St. 3010, central Gulf of California).

**Distribution.** Sitka, Alaska, USA into the Gulf of California northeast of Isla Santa Cruz, Baja California Sur, Mexico south to Peru–Chile Trench, Lima, Peru; western Pacific from off Mys Olyutorsky to Honshu, Japan. 100–5,379 m (Coan and Valentich-Scott 2012).

**Environmental conditions.** DO, 0.08 ml/l; T, 6.15° C; S, 34.55; sed., 11.04% Sa, 82.96% Si, 5.99% Cl.

**Remarks.** Unfortunately, no living specimens of this widely distributed species were collected during this cruise.

**Order Nuculanida**

**Superfamily Nuculanoidea**

**Family Nuculanidae H. Adams & A. Adams, 1858 (1854)**

***Jupiteria callimene* (Dall, 1908)**

Fig. 1d

**Material examined.** St. 6 (24° 13' 42" N, 112° 09' 24" W), July 29, 2012, 296–312 m depth, benthic sledge, 2 specimens (ICML–EMU–10978) and 2 specimens (SBMNH 235542).

**Type locality.** East of "Cape Mala", Gulf of Panama (Albatross St. 3396).

**Distribution.** Western Baja California, Mexico to Panama (this contribution; Coan and Valentich-Scott 2012). 183–3200m (Coan and Valentich-Scott 2012).

**Environmental conditions.** DO, 0.05 ml/l; T, 10.6°C; S, 34.7; sed., 14.88% Sa, 79.95% Si, 5.18% Cl.

**Remarks.** The specimens examined are somewhat intermediate between *J. lobula* (Dall, 1908), from Mexico to El Salvador, and *J. callimene* (Dall, 1908), from Costa Rica and Panama. In examining the type specimen of each species, we found our specimen had a heavy hinge plate, with wider, more numerous, and more closely packed teeth. This clearly indicates that it is aligned with *J. callimene*. This new record extends the distribution of *J. callimene* by about 16°42' of latitude to the north to western Mexico. The depth range of the Baja California specimens examined is within the known range of the species (see Table 1).

***Jupiteria pontonia* (Dall, 1890)**

Fig. 2a

**Material examined.** St 8 (24° 25' 48" N, 112° 38' 06" W), July 30, 2012, 1 specimen, 1212–1235 m, benthic sledge (ICML–EMU–9984).

**Type locality.** Near Galapagos Islands (United States Fish Commission Sts. 2807 and 2808).

**Distribution.** Santa Barbara and San Diego, California, USA; southern Gulf of California, Mexico to the Gulf of Panama; Galapagos Islands. 1100–3000 m (Zamorano et al. 2007, Coan and Valentich-Scott 2012).

**Environmental conditions.** DO, 0.65 ml/l; T, 3.44°C; S, 34.6; sed., 29.59% Sa, 61.92% Si, 8.48% Cl.

**Remarks.** The material examined represents new sampling localities and the first records from off the west coast of Baja California, Mexico, thus filling the previous distributional gap between southern California and the Gulf of California records.

**Nuculana cf. hamata (Carpenter, 1864)**

Fig. 2b

**Material examined.** St. 1 (26° 30' 42" N, 113° 56' W), August 4, 2015, 1 empty specimen, 750–850 m depth, box core (ICML–EMU–9977).

**Type locality.** Catalina Island, California, USA.

**Distribution.** Forrester Island, Alaska, USA into the Gulf of California as far north as Isla Angel de la Guarda, Baja California, Mexico. 20–1100 m (Coan and Valentich-Scott 2012).

**Environmental conditions.** DO, 0.11 ml/l; T, 5.75°C; S, 34.54. 18.69% Sa, 67.94% Si, 13.37% Cl.

**Remarks.** The collected shell specimen is badly damaged, hence the identification is tentative. The specimen falls into a large species group (see illustrations in Coan and Valentich-Scott 2012) and potentially several new species need to be described in this poorly studied complex.

**Family Neilonellidae Schileyko, 1989**

***Neilonella mexicana* (Dall, 1908)**

**Fig. 2c**

**Material examined.** St. 24 (27° 05' 42" N, 114° 35' 30" W), August 1, 2015, 3 specimens, box core, 772–786 m depth (ICML–EMU–9978).

**Type locality.** Off Mexican coasts (Albatross St. 3418, off Guerrero, Mexico).

**Distribution.** Farallon Islands, California, USA, into the Gulf of California, as far north as Isla Santa Cruz, Baja California Sur, Mexico, to Panama. 780–3060 m (Coan and Valentich-Scott 2012).

**Environmental conditions.** DO, 0.12 ml/l; T, 5.24°C; S, 34.53; sed., 35.53% Sa, 56.52% Si, 7.95% Cl.

**Remarks.** One of the two species of this genus previously recorded from tropical West America. The other species being *Neilonella atossa* (Dall, 1908), known from California to the Gulf of California, and Punta Agua, Peru.

***Neilonella ritteri* (Dall, 1916)**

**Fig. 2d**

**Material examined.** St. 23 (27° 08' 11" N, 114° 32' 54" W), August 1, 2015, 1 specimen, 530–625 m, benthic sledge (ICML–EMU–9981).

**Type locality.** Off La Jolla, California, USA (United States Fish Commission St. 4325).

**Distribution.** Santa Barbara, California, USA to west of Isla Altamura, Sinaloa, SW Gulf of California, México. 366 to 860 m (Zamorano et al. 2007, Coan and Valentich-Scott 2012).

**Environmental conditions.** DO, 0.068 ml/l; T, 6.44°C; S, 34.47; sed., 46.62% Sa, 46.56 % Si, 6.82% Cl.

**Remarks.** This newly collected material represents a new sampling locality and the first record from off the west coast of Baja California, Mexico, thus filling a distributional gap between southern California and the Gulf of California records.

**Order Pectinida**

**Superfamily Pectinoidea**

**Family Pectinidae Rafinesque, 1815**

***Delectopecten vancouverensis* (Whiteaves, 1893)**

Fig. 2e

**Material examined.** St. 23 (27° 08' 11" N, 114° 32' 54" W), August 1, 2015, 2 specimens, 530–625 m, benthic sledge (ICML–EMU–10975).

**Type locality.** Quatsino Sound, Vacouver Island, Bristish Columbia, Canada.

**Distribution.** Alaska, USA, to Isla San Benito and Isla Cedros, Pacific coast of Baja California; in the Gulf of California off Guaymas, Sonora, Mexico; Kamchatka to northern Japan. 27–4100 m (Coan and Valentich-Scott 2012).

**Environmental conditions.** DO, 0.068 ml/l; T, 6.44°C; S, 34.47; sed., 46.62% Sa, 46.56 % Si, 6.82% Cl.

**Remarks.** The material examined represents a new sampling locality for this species.

**Order Limida**

**Superfamily Limoidea**

**Family Limidae Rafinesque, 1815**

***Acesta sphoni* (Hertlein, 1963)**

**Fig. 3a**

**Material examined.** St. 1 (23° 18' 40" N, 111° 19' 37" W), August 4, 2015, 1 specimen, 750–850 m, benthic sledge (ICML–EMU–9978). St. 20 (26° 30' 42" N, 113° 56' W), August 2, 2015, 10 specimens, 540–568 m depth, benthic sledge (ICML–EMU–9979). St. 23 (27°08'11"N, 114°32'54"W), August 1, 2015, 11 specimens, 530–625 m, benthic sledge (ICML–EMU–9980, 9982, 9983, 10033).

**Type locality.** Between Santa Catalina and Santa Barbara Island, California, USA.

**Distribution.** San Juan Bank, California, USA, to Gulf of California, Mexico. 457–850 m (Clague et al. 2012; this contribution).

**Environmental conditions.** DO, 0.068–0.15 ml/l; T, 5.75–8.38°C; S, 34.47–34.54.; sed., 18.69–46.62% Sa, 45.16–67.94 % Si, 6.82–13.37% Cl.

**Remarks.** The material of ICML–EMU–9978 is about 15 mm height and probably represents a juvenile of this species. Two species of *Acesta* have been reported for tropical West America by Coan and Valentich-Scott (2012): *A. agassizii* (Dall, 1902), from the Gulf of California to Panama and Islas Galápagos, and *A. diomedae* (Dall, 1908), from off the Islas Galápagos. The other species previously known in the area, *A. sphoni* and *A. mori*, both from northern Oregon to southern California, were recently reported by Walz et al. 2014) from the Gulf of California for the first time. Walz et al. 2014 also provided greatest depth records for both species. *Acesta sphoni* occurs at shallower depths than *A. mori*, in warmer water with less oxygen (Walz et al. 2014). The material examined herein, however, was collected in depths from 540 to 850 m, thus increasing the maximum known depth for this species by ca 300 m (see Table 1).

**Limatula cf. saturna F.R. Bernard, 1978**

**Fig. 3b**

**Material examined.** St 17 (26°20'24"N, 114°13'07"W), July 31, 2015, 1 specimen, 2285 m, box core (ICML–EMU–9985).

**Type locality.** Off Saturna Island, Strait of Georgia, British Columbia.

**Distribution.** Albatross Bank, Kodiak Island, Alaska, USA to Cabo San Lucas, Baja California Sur and Isla Carmen as far north as Bahia de Los Angeles, Baja California and Bahia San Carlos, Sonora, Mexico. 20–675 m (Zamorano et al. 2007; Coan & Valentich-Scott 2012).

**Environmental conditions.** DO, 1.62 ml/l; T, 2.15°C; S, 34.67; sed., 2.82% Sa, 80.97% Si, 16.21% Cl.

**Remarks.** Two species are reported from tropical West America: *L. saturna* F.R. Bernard, 1978, from Alaska to the Gulf of California, and *L. similaris* (Dall 1908), also from Alaska to the Gulf of California, but extending to Central America and also present in Galapagos Islands. The specimens examined are similar to the holotype of *L. saturna*. In comparison to the type our specimens are more inflated, wider, and have more reduced ears. The depth range of *L. saturna* is from 20–675 m (Table 1). Our specimens identified as L. cf. saturna were collected in much deeper water than previous reports. This, combined with the unusual morphology, reinforces the idea that it might represent a new species.

**Order Lucinida**

**Superfamily Lucinoidea**

**Family Lucinidae J. Fleming, 1828**

***Lucinoma aequizonatum* (Stearns, 1890)**

**Fig. 3c**

**Material examined.** St 7 (24° 27' 06" N, 112° 27' W), July 27, 2015, 17 specimens, 528–540 m, benthic sledge (ICML–EMU–9986). St 5D (23° 16' 58" N, 110° 20' 42" W), August 5, 2015, 2 empty specimens, 650–665 m, benthic sledge (ICML–EMU–9987).

**Type locality.** Off Santa Barbara Islands, California.

**Distribution.** Santa Barbara Channel, California, USA, into the Gulf of California, as far north as northwest of Isla Santa Cruz, Mexico to Chile. 400–1310 m (Zamorano et al. 2007, Coan and Valentich-Scott 2012).

**Environmental conditions.** DO, 0.06–0.08 ml/l; T, 6.15–8.49°C; S, 34.55–34.59; sed., 11.04% Sa, 82.96% Si, 5.99% Cl.

**Remarks.** The genus *Lucinoma* is represented in the tropical West Pacific by three species: *L. aequizonatum*, from California to the Gulf of California, *L. annulatum* (Reeve, 1850), reported from a very wide latitudinal range, from Japan and Alaska south to the Gulf of California and Costa Rica, and *L. heroica* (Dall, 1901), known from the Gulf of California and off Pisco, Peru. The material reported by Zamorano and Hendrickx (2012)as *L. heroica* belongs to *L. aequizonatum* (see depth range for this species in Table 1, partly obtained from Zamorano and Hendrickx (2012). *Lucinoma aequizonatum* is an extremophile and is characteristic of the most sulfide–rich, methane–rich, and oxygen–poor environments of modern continental margins (Moffitt et al. 2015). The material examined, although very similar to *L. aequizonatum* in shell morphology, might represent an undescribed species (John Taylor, pers. comm., December, 2013).

**Clade Septibranchia**

**Superfamily Cuspidarioidea**

**Family Cuspidariidae**

***Cardiomya planetica* (Dall, 1908)**

**Fig. 4a**

**Material examined.** St. 20 (26° 30' 42" N, 113° 56' W), August 2, 2015, 1 specimen, 540–568 m depth, box corer (ICML–EMU–9988) and 6 specimens, benthic sledge (ICML–EMU–10977).

**Type locality.** Off San Diego, California, USA (Albatross St. 2925).

**Distribution.** Pribilof Islands, USA, to Cedros Island and in the Gulf of California, Mexico, to the Gulf of Panama; Galapagos Islands. Also reported from Japan. 25–3,000 m (Zamorano et al. 2007, Coan and Valentich-Scott 2012).

**Environmental conditions.** DO, 0.15 ml/l; T, 8.38°C; S, 34.51; sed., 47.08% Sa, 45.16% Si, 7.75% Cl.

**Remarks.** This genus is represented by five species in the region. This new sampling confirms the presence of this species further south along the Baja California Peninsula (ca 2 degrees of latitude).

***Luzonia chilensis* (Dall, 1890)**

**Fig. 4b**

**Material examined.** St. 20 (26° 30' 42" N, 113° 56' W), August 2, 2015, 1 specimen, 540–568 m depth, benthic sledge (ICML–EMU–9989).

**Type locality.** Off SW coast of Chile (United States Fish Commission St. 2791).

**Distribution.** Destruction Island, Washington, USA, into the Gulf of California as far north as Guaymas Basin, Sonora, Mexico, to southern Chile. 100 to 1875 m (Coan and Valentich-Scott 2012).

**Environmental conditions.** DO, 0.15 ml/l; T, 8.38°C; S, 34.51; sed., 47.08% Sa, 45.16% Si, 7.75% Cl.

**Remarks.** This is the only species of this genus collected in the region. The material examined represents a new sampling locality and the first record from off the west coast of Baja California, Mexico, thus filling the distributional gap between southern California and the Gulf of California records.

**Superfamily Poromyoidea**

**Family Poromyidae Dall, 1886**

***Dermatomya mactroides* (Dall, 1889)**

Fig. 4c

**Material examined.** St. 24 (27° 05' 42" N, 114° 35' 30" W), August 1, 2015, 1 specimen, 772–786 m depth (ICML–EMU–9991).

**Type locality.** Off coasts of Ecuador.

**Distribution.** From Santa Cruz Island, California, USA, to east of San José Island, Baja California Sur, Gulf of California, Mexico, and to southern Chile. 120 to 1185 m (Zamorano et al. 2007; Coan and Valentich-Scott 2012).

**Environmental conditions.** DO, 0.12 ml/l; T, 5.24°C; S, 34.53; sed., 35.53% Sa, 56.52% Si, 7.95% Cl.

**Remarks.** This is the only species of this genus collected in the region. The material examined represents a new sampling locality and the first record from off the west coast of Baja California, Mexico, thus filling the distributional gap between southern California and the Gulf of California records.

**Superfamily Verticordioidea**

**Family Verticordiidae Stoliczka, 1870**

***Policordia* sp.**

Fig. 4d

**Material examined.** St. 23 (27°08'11"N, 114°32'54"W), 1 specimen, 530–625 m, benthic sledge (ICML–EMU–9990).

**Environmental conditions.** DO, 0.068 ml/l; T, 6.44°C; S, 34.47; sed. 46.62% Sa, 46.56% Si, 6.82% Cl.

**Remarks.** Shells of *Policordia* are very close to shells of species of *Dallicordia* and can be separated only through a detailed examination of soft parts. Based on a careful examination of internal anatomy (i.e., gills structure, tentacles of exhalant siphon) of the small, unique specimen available, we concluded that it belongs to the genus *Policordia*. However, there are no records for this genus in the eastern Pacific south of 44°N. Material representing a new *Policordia* from southern California is currently being studied (E. Krylova, pers. comm., September 2015) and our material could eventually prove to belong to the same species.

## Discussion

**Figure 5.**
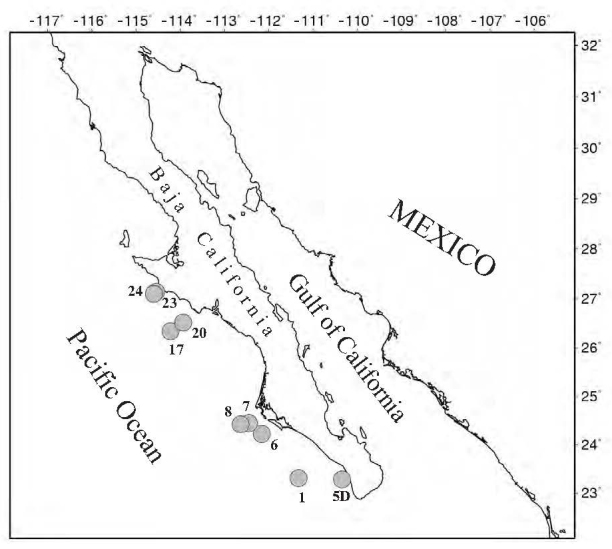
Distribution of sampling stations where specimens of bivalves were collected during the TALUD XV survey off the western coast of the Baja California Peninsula.

The material collected during the TALUD XV brings interesting information on the very rich deep-water mollusk fauna occurring off the west coast of the peninsula of Baja California. Specimens obtained also came from a wide latitudinal range (Fig. 5). Two species were recorded for the first time off western Mexico, with a distributional range increase of 16-20 degrees to the north (i.e., *Ennucula panamina* and *Jupiteria callimene*). In addition, five species have been collected for the first time off the west coast of Baja California Peninsula (i.e., *Ennucula taeniolata*, *Neilonella ritteri*, *Lucinoma aequizonatum*, *Luzonia chilensis*, and *Dermatomya mactroides*). New localities significantly filling the gap of their previously known distribution range were presented for four species (Table 1). As in the case of other invertebrates, diversity and distribution of deep-water mollusks is under the influence of many factors. Temperature, dissolved oxygen, sediment types and organic matter content in sediments are among the most important (Levin et al. 2001). In an area where the Oxygen Minimum Zone (ZMO) is particularly extended (Serrano 2012), dissolved oxygen values are critical for the survival of sedentary species. Some species of mollusks (e.g., *Lucinoma* spp.) are known to tolerate extremely low oxygen concentrations and are able to survive in severe hypoxic conditions (Zamorano and Hendrickx 2012; Vaquer-Sunyer and Duarte 2008). Except for two samples (i.e., *Jupiteria pontonia* and Limatula cf. saturna), the material collected during the TALUD XV cruise was obtained in a very narrow oxygen range: 0.05 to 0.15 ml O_2_/l. Both *A. sphoni* and *L. aequizonatum* have been previously reported as occurring repeatedly in very low oxygen conditions (Clague et al. 2012; Taylor and Glover 2010).

Although the general depth range sampled during the TALUD XV cruise was 296 to 2285 m, it is surprising that samples of bivalves were generally obtained in a very narrow range, i.e., from 528 and 850 m: 83% of the species and 92% or the total number of specimens. Number of species found in each station was low (1 to 6). Although the numbers of species and specimens obtained were few, five and four species were collected in stations 23 and 20, respectively (Table 1). This is an interesting find considering that so little is known about deep-water mollusks communities composition below the OMZ in hypoxic conditions and about their adaptations and tolerance to oxygen deficiency.

Due to a general lack of information and of comparative material of deep-water bivalves in this region of the world, several species could not be properly identified and some might represent new species. This demonstrates that this fauna is poorly known and further surveys will without any doubts bring much needed data on deep-water bivalves of the Mexican Pacific.

## Conclusions

A total of 17 species of deep-water bivalves were collected during the survey. The collection allows for the addition of new information related to the bathymetric and geographic distribution of these species. Some specific taxonomic issues remained to be solved, in particular in what concerns the identity of Ennucula panamina and E. taeniolata. Other material in need of a thorough review are Nuculana cf. hamata and *Limatula* cf. *saturna*. Besides, important ecological information was available for all species reported, including data on dissolved oxygen, temperature, salinity and sediments composition which are usually lacking for deep-water mollusks fauna. The studied bivalvia fauna is specifically associated with the lower boundary of the Oxygen Minimum Zone which is one of the major oceanographic characteristics in the eastern Pacific.

## Figures and Tables

**Figure 1a.**
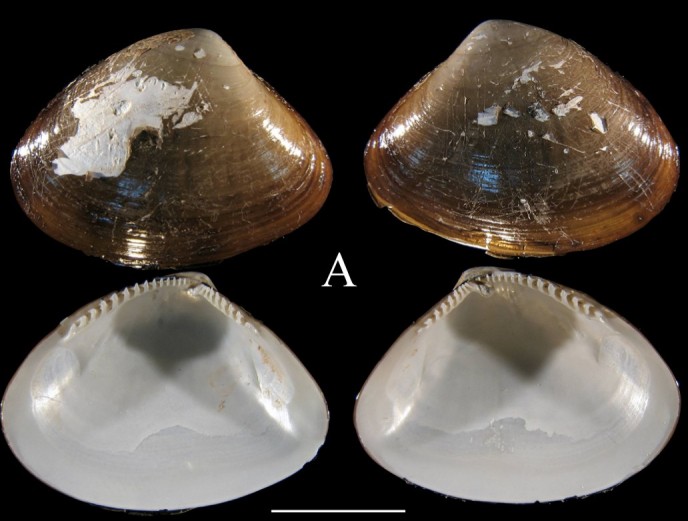
A. *Ennucula panamina*, ICML–EMU 10974 (sb = 1cm).

**Figure 1b.**
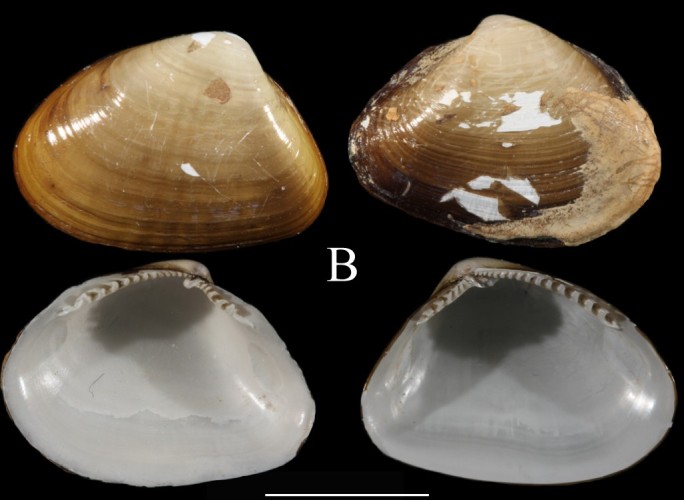
B. *Ennucula taeniolata*, ICML–EMU 9974 (sb = 1 cm).

**Figure 1c.**
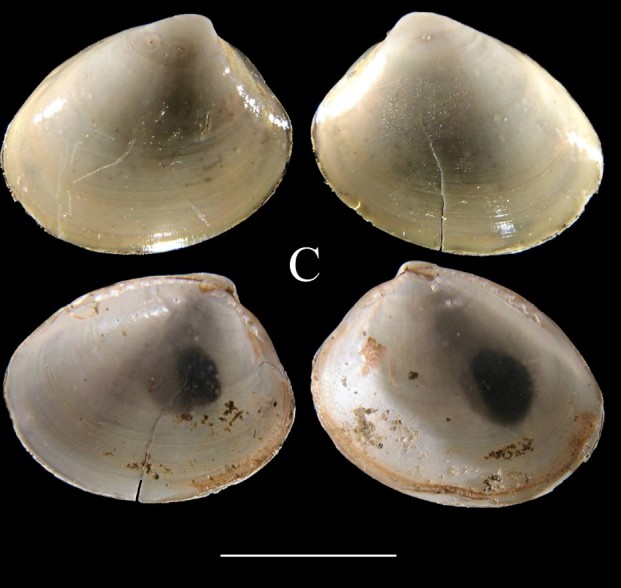
C. *Ennucula tenuis*, ICML–EMU 9975 (sb = 0.5 cm).

**Figure 1d.**
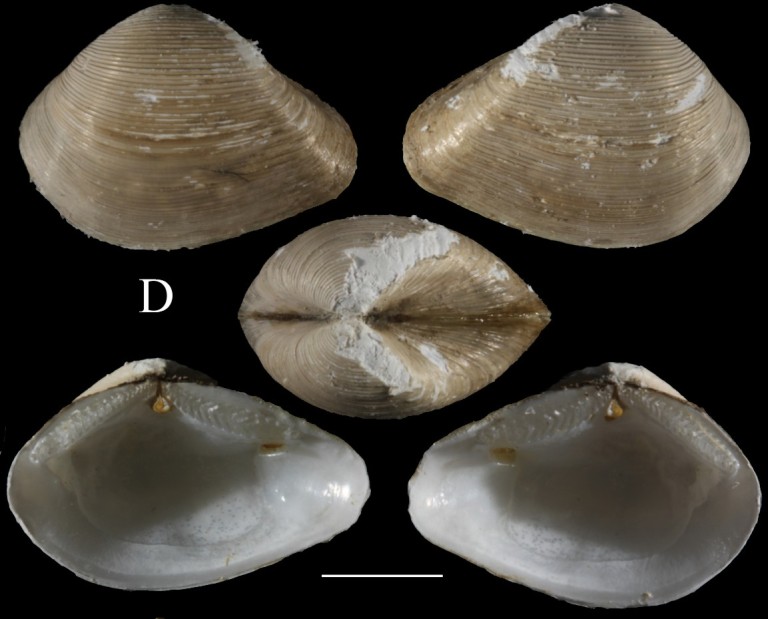
D. *Jupiteria callimene*, SBMNH 235542 (sb = 0.5 cm).

**Figure 1e.**
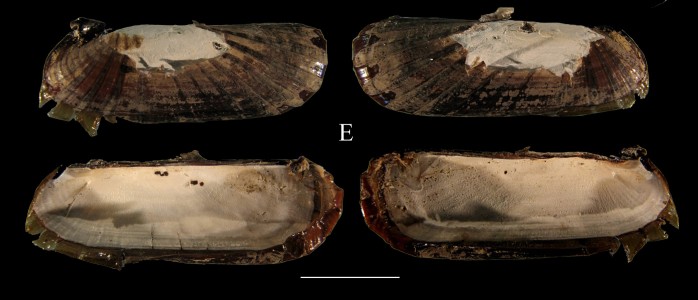
E. *Acharax johnsoni* ICML–EMU 9976 (sb = 1 cm).

**Figure 2a.**
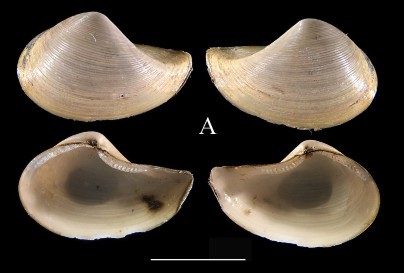
A. *Jupiteria pontonia*, ICML–EMU 9984 (sb =1cm).

**Figure 2b.**
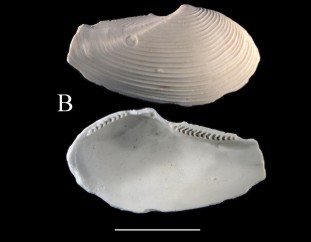
B. Nuculana cf. hamata, ICML–EMU 9977 (sb = 0.5 cm).

**Figure 2c.**
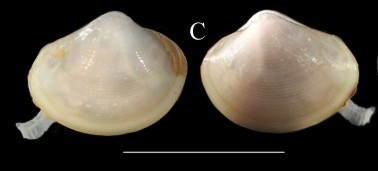
C. *Neilonella mexicana*, ICML–EMU 9978 (sb = 0.5 cm).

**Figure 2d.**
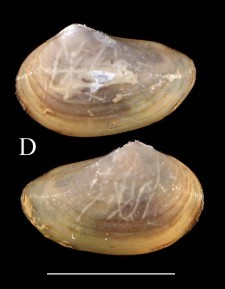
D. *Neilonella ritteri*, ICML–EMU 9981 (sb = 0.5 cm).

**Figure 2e.**
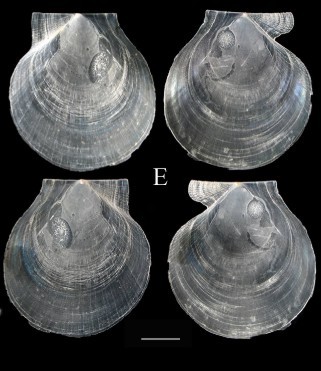
E. *Delectopecten vancouverensis*, ICML–EMU 10975 (sb = 0.4 cm).

**Figure 3a.**
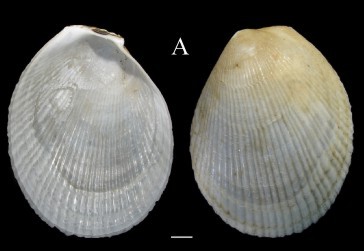
A. *Acesta sphoni*, ICML–EMU 9978 (sb = 1 cm).

**Figure 3b.**
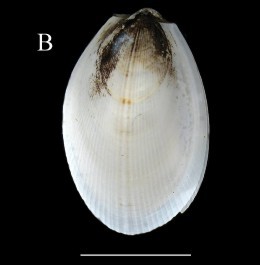
B. Limatula cf. saturna, ICML–EMU 9985 (sb = 1cm).

**Figure 3c.**
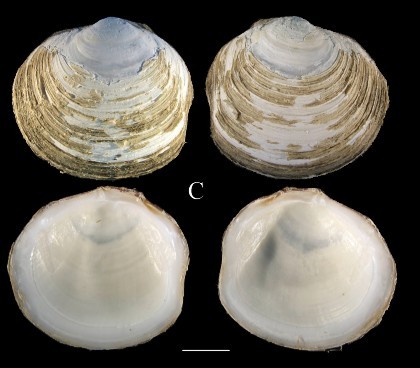
C. *Lucinoma aequizonatum*, ICML–EMU 9986 (sb = 0.5 cm).

**Figure 4a.**
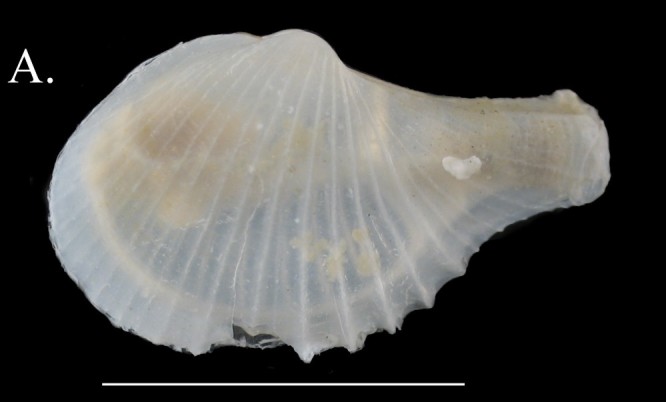
A. *Cardiomya planetica*, ICML–EMU 10977 (sb = 0.5 cm).

**Figure 4b.**
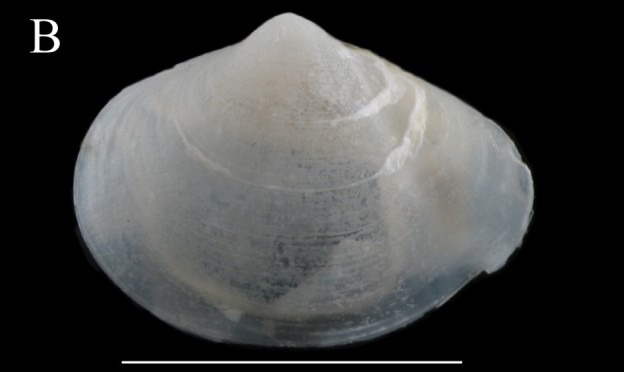
B. *Luzonia chilensis*, ICML–EMU 9989 (sb = 0.5cm).

**Figure 4c.**
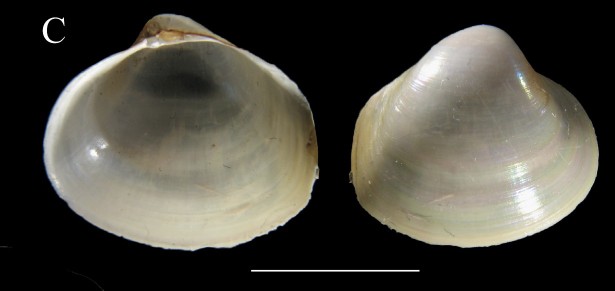
C. *Dermatomya mactroides*, ICML–EMU 9991 (sb = 1cm).

**Figure 4d.**
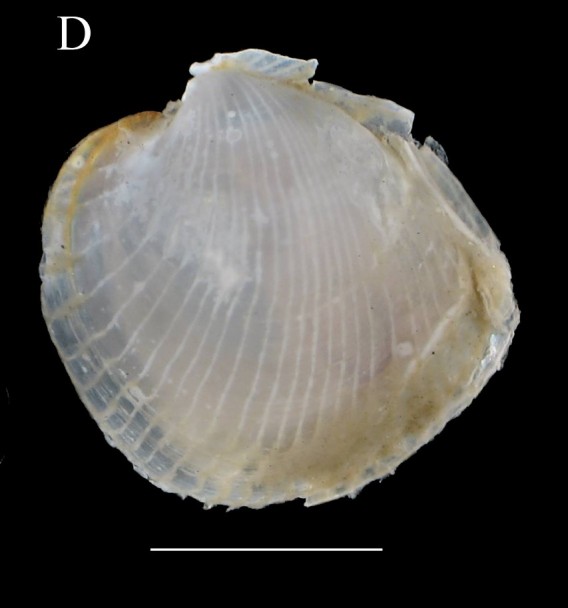
D. *Policordia* sp., ICML–EMU 9990 (sb = 0.3 cm).

**Table 1.** Synthesis of the material of bivalves collected during the TALUD XV cruise, including species per station, station depth, number of specimens and depth previously reported for each species (see text for species depth range references). D, dead specimen; L, live specimen. See Fig. 5 for species distribution. ^(1)^ Record extension to the north; ^(2)^ first record off western Baja California; ^(3)^ new localities.

**Station**	**Depth (m)**	**Species**	**Specimens**	**Known depth (m)**
23	530–625	*Ennucula panamina* ^(1)^	5 (L) 1 (D)	550–3058
		*Ennucula taeniolata* ^(2)^	1 (L)	902–1275
		*Neilonella ritteri* ^(2)^	1 (L)	366–860
		*Delectopecten vancouverensis* ^(3)^	2 (L)	27–4100
		*Acesta sphoni*	11 (L)	457–549
		*Policordia* sp.	1 (L)	450–3570
20	540–568	*Ennucula panamina* ^(1)^	14 (L)	550–3058
		*Acesta sphoni*	10 (L)	457–549
		*Cardiomya planetica* ^(3)^	7 (L)	25–3000
		*Luzonia chilensis* ^(2)^	1 (L)	100–1875
24	772–786	*Ennucula tenuis* ^(3)^	1 (L)	20–1450
		*Neilonella mexicana*	3 (L)	780–3060
		*Dermatomya mactroides* ^(2)^	1 (L)	120–1185
5D	650–665	*Acharax johnsoni*	5 (D)	100–5379
		*Lucinoma aequizonatum* ^(2)^	2 (L)	400–1310
6	296–312	*Jupiteria callimene* ^(1)^	4 (L)	183–3200
8	1212–1235	*Jupiteria pontonia* ^(3)^	1 (L)	1100–3000
1	750–850	Nuculana cf. hamata	1 (L)	20–2100
		*Acesta sphoni*	1 (L)	457–549
17	2285	Limatula cf. saturna	1 (L)	20–675
7	528–540	*Lucinoma aequizonatum* ^(2)^	17 (L)	400–1310

## Project

Biodiversidad y recursos pesqueros en las aguas profundas de la costa oeste de la peninsula de Baja California.

## Hosting institution

Unidad Académica Mazatlán, Instituto de Ciencias del Mar y Limnología, Universidad Nacional Autónoma de México

## Project

Biodiversidad y recursos pesqueros en las aguas profundas de la costa oeste de la peninsula de Baja California.

## Hosting institution

Unidad Académica Mazatlán, Instituto de Ciencias del Mar y Limnología, Universidad Nacional Autónoma de México
